# Long-Term Impact of Chronic Obstructive Pulmonary Disease and Atrial Fibrillation on Post-Acute Myocardial Infarction Long-Term All-Cause Mortality: Insights from the SAMI III Project

**DOI:** 10.3390/jcm14165907

**Published:** 2025-08-21

**Authors:** Arthur Shiyovich, Harel Gilutz, Keren Skalsky, Ygal Plakht

**Affiliations:** 1 Division of Cardiovascular Medicine, Department of Medicine, Brigham and Women’s Hospital, Harvard Medical School, 75 Francis Street, Boston, MA 02115, USA; 2 Faculty of Health Sciences, Ben-Gurion University of the Negev, Beer-Sheva 8410501, Israel; gilutzh@bgu.ac.il (H.G.); plakht@bgu.ac.il (Y.P.); 3 Department of Cardiology, Rabin Medical Center, Petach Tikva 4941492, Tel Aviv University, Tel Aviv 6139001, Israel; kskalsky@gmail.com; 4 Department of Emergency Medicine, Soroka University Medical Center, Beer-Sheva 84101, Israel

**Keywords:** acute myocardial infarction, chronic obstructive pulmonary disease, atrial fibrillation, mortality

## Abstract

**Background:** Chronic obstructive pulmonary disease (COPD) and atrial fibrillation (AF) are common comorbidities in patients with acute myocardial infarction (AMI) and are associated with adverse cardiovascular outcomes. However, the impact of their coexistence on long-term post-AMI outcomes remains unclear. This study aimed to investigate the long-term effects of COPD and AF on AMI survivors. **Methods:** This retrospective cohort study analyzed data from consecutive AMI hospitalizations between 1 January 2002 and 31 October 2017. Patients were categorized into four groups based on the presence or absence of COPD and AF. The primary outcome was all-cause mortality up to 10 years post-discharge. Multivariate survival models were used to assess independent associations. **Results:** A total of 15,449 AMI survivors (mean age 66 ± 14 years, 30% female) were included, of whom 1386 (8.9%) had COPD, 2547 (16.5%) had AF, and 376 (2.4%) had both conditions. Over a median follow-up of 7.7 (IQR 3.3–10) years, 44.7% of the patients died. COPD (AdjHR = 1.89, 95% CI: 1.74–2.05), AF (AdjHR = 1.39, 95% CI: 1.31–1.48), and coexistence of both conditions (AdjHR = 1.82, 95% CI: 1.61–2.04) were associated with an increased risk for mortality (*p* < 0.001 for each). However, in patients with both conditions, the mortality risk was comparable to that of COPD alone. **Conclusions:** While both COPD and AF are associated with increased long-term mortality after AMI, COPD appears to be the primary independent driver of this risk. These findings underscore the need for proactive screening and individualized management in this high-risk population.

## 1. Introduction

Chronic obstructive pulmonary disease (COPD) and atrial fibrillation (AF) are among the most common comorbidities among patients with coronary artery disease (CAD) in general and acute myocardial infarction (AMI) in particular [1,2,3,4,5,6,7]. Moreover, there is a notable interaction between these two conditions, particularly in the elderly. Approximately 8% of patients with COPD also have AF, while around 13% of individuals with AF are diagnosed with COPD [8]. This interplay has been referred to in the literature as “AF-COPD syndemic” [9,10,11,12]. Both conditions are associated with a higher prevalence of atherosclerosis and adverse cardiovascular outcomes [4,8,10,13,14,15,16]. Furthermore, COPD was found to be independently associated with an increased incidence of AF after AMI [17]. The coexistence of AF and COPD has been linked to more complex management, suboptimal treatment, worse post-cardiovascular procedural outcomes, and variations in cardiovascular drug prescriptions due to safety concerns [4,8,10,18,19]. Additionally, factors such as inflammation, hypoxia-mediated mechanisms, autonomic dysfunction, beta-2 agonist use, and increased atrial pressure contribute to their intricate interplay and potential negative effects in patients with CAD [5,13]. However, the impact of their coexistence on post-AMI outcomes is yet to be elucidated. The aim of this study was to investigate the long-term effects of COPD and AF in patients following AMI using a large AMI registry.

## 2. Methods

### 2.1. Study Population and Outcomes

Our study is a retrospective exploratory analysis of the Soroka University Medical Center registry (Soroka Acute Myocardial Infarction [SAMI] project), including consecutive patients with acute myocardial infarction (AMI) hospitalized between 1 January 2002 and 31 October 2017 [20,21]. The study included adult Israeli citizens (≥18 years old) who were hospitalized with AMI and survived the index event. Patients with atrial flutter were excluded. For individuals with multiple AMI admissions during the study period, only the last occurrence was considered. The primary outcome was all-cause mortality up to 10 years post-discharge or until 31 July 2023, whichever occurred first.

This study adhered to the Declaration of Helsinki and was approved by Soroka’s Institutional Review Board (approval number SOR-0319–16), with informed consent waived due to its retrospective design.

### 2.2. Data Collection and Definitions

As previously described [20,21], clinical data were retrieved from an electronic medical record, in which baseline comorbidities were identified by International Classification of Diseases, Ninth Revision, Clinical Modification (ICD-9-CM) codes, as documented in real time by the treating medical team and according to prespecified criteria. Death events were obtained from the Israeli Ministry of the Interior Population Registry.

The diagnosis of acute myocardial infarction (AMI) was determined using International Classification of Diseases, Ninth Revision, Clinical Modification (ICD-9-CM) codes: ST-elevation AMI (STEMI) (410.0*–410.6*) and non-ST-elevation AMI (NSTEMI) (410.7*–410.9*). Disease classifications and interventions were categorized based on ICD-9-CM discharge codes. Obstructive CAD was defined as the presence of ≥70% stenosis in a coronary vessel, as assessed by angiography (“*” indicates all the codes in ICD-9 category or subcategory).

COPD was identified using ICD-9-CM codes 490–496. AF was defined by diagnostic code 427.31, based on discharge records. Transthoracic echocardiograms were conducted and interpreted in accordance with established guidelines, including those of the American Society of Echocardiography. Severe LV dysfunction was defined as an ejection fraction of <30% on the first echocardiogram performed during hospitalization [22].

The cohort was categorized based on the presence or absence of COPD and AF. Subsequently, four groups were created as follows: **Group 1:** No COPD or AF (COPD−, AF−). **Group 2:** With COPD, but without AF (COPD+, AF−). **Group 3:** No COPD but with AF (COPD−, AF+). **Group 4:** With both COPD and AF (COPD+, AF+).

### 2.3. Statistical Analysis

Statistical analysis was performed using the Statistical Package for the Social Sciences (SPSS), version 29 (IBM Corporation, Armonk, NY, USA). Variables were reported as frequencies and percentages, medians and interquartile ranges (IQRs), or means and standard deviations (SD), and compared using Pearson’s chi-square, Student’s *t*, and analysis of variance (ANOVA) tests. Additionally, Bonferroni corrections for Pearson’s chi-square and for ANOVA tests (paired comparisons) were applied.

The time-dependent probability and cumulative mortality were assessed by the Kaplan–Meier survival approach and compared using the log-rank test with a Bonferroni correction. Independent associations with the risk for all-cause death were evaluated by Cox proportional hazard regression analyses at univariable and multivariable levels. In these models, Group 1 (COPD−, AF−) served as the reference group. Multivariable analysis incorporated baseline variables demonstrating a *p*-value of <0.1 at the univariable stage using a stepwise approach. Lastly, an interaction analysis was undertaken, in which the variable of multiplication (COPD × AF) was included in the model. Missing data (i.e., echocardiography and coronary angiography results) were handled by creating a separate category within the respective parameters for cases with missing values. The results of the models were presented as hazard ratios (HR)/adjusted hazard ratios (adjHR) with their 95% confidence intervals (CI). Statistical significance required a two-sided *p*-value of <0.05.

## 3. Results

### 3.1. Study Population and Groups

A total of 15,449 patients who met the inclusion and exclusion criteria were included in the final cohort (mean age: 65.8 [SD = 13.9] years, 29.5% female). The patients’ cohort consisted of 1386 (8.9%) patients with COPD, 2547 (16.5%) patients with AF, 376 (2.4%) patients with both conditions, and 11,892 (76.9%) patients with neither COPD nor AF (see Supplemental Figure S1).

### 3.2. Patient Characteristics

Overall, patients with COPD and those with AF were older and presented with multimorbidity compared to those without these conditions. Table 1 outlines the baseline characteristics of the study cohort across the study groups. The baseline characteristics of the patients, stratified by COPD and AF status, are presented in Supplemental Table S1A,B. Post-AMI patients with COPD, AF, or both were older and had a higher prevalence of cardiovascular comorbidities and risk factors. However, a family history of ischemic heart disease was more common in the groups of patients with no COPD and with no AF. Smoking was significantly more prevalent among those with COPD, regardless of AF status. STEMI was less frequent than NSTEMI in patients with AF, COPD, or both, and hospital length of stay was longer for these groups. Multivessel CAD, severe left ventricular dysfunction, and certain in-hospital complications—particularly mechanical ventilation and blood transfusion—were more common in patients with COPD or AF, especially when both conditions coexisted. Patients with AF, COPD, or both conditions were in particular less likely to undergo revascularization through percutaneous coronary intervention (PCI) or coronary artery bypass graft (CABG).

### 3.3. Follow-Up and Primary Outcome

Over a median follow-up of 7.7 years (IQR 3.3–10 years), a total of 6910 patients (44.7%) died, with a cumulative mortality rate of 0.468. Among patients with COPD, the mortality rate was 76.0% (1053/1386), with a cumulative mortality of 0.790, whereas patients without COPD had a significantly lower mortality rate of 41.6% (5857/14,063) and a cumulative mortality of 0.437 (*p* < 0.001). Similarly, patients with AF experienced a mortality rate of 75.8% (1930/2547) and a cumulative mortality of 0.781, in contrast to those without AF, who had a mortality rate of 38.6% (4980/12,902) and a cumulative mortality of 0.406 (*p* < 0.001) (see Supplemental Figure S2).

**Figure 1:**
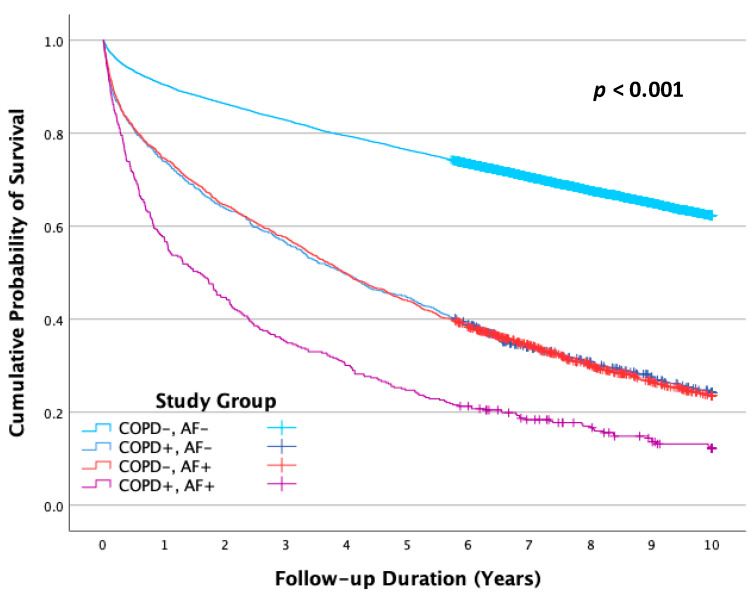
Cumulative survival functions for post-acute myocardial infarction all-cause mortality throughout the follow-up period up to 10 years by study group. AF—Atrial fibrillation, COPD—Chronic obstructive pulmonary disease.

Mortality rates varied significantly across study groups (*p* < 0.001), with 4249 deaths (35.7%) in Group 1 (COPD−, AF−), 731 deaths (72.4%) in Group 2 (COPD+, AF−), 1608 deaths (74.1%) in Group 3 (COPD−, AF+), and 322 deaths (85.6%) in Group 4 (COPD+, AF+). Cumulative mortalities by the study group were 0.377, 0.758, 0.765, and 0.878 for Groups 1, 2, 3, and 4, respectively (*p* < 0.001) (see Figure 1). Notably, there was significantly higher mortality in patients with either COPD or AF (Groups 2 and 3) compared to patients with none of these conditions (Group 1) (*p* < 0.001 for each). The highest mortality was observed in Group 4 when both conditions coexisted (*p* < 0.001, compared with Group 1). When only one of the conditions was present, mortality rates were very similar (*p* = 0.922, in comparison between Groups 2 and 3).

### 3.4. COPD, AF, and the Risk for Mortality

In the univariable analysis, both COPD and AF were significantly associated with increased mortality risk. COPD was associated with HR of 2.772 (95% CI: 2.595–2.961, *p* < 0.001). Similarly, AF demonstrated an even higher HR of 2.915 (95% CI: 2.765–3.073, *p* < 0.001). Furthermore, univariable analysis revealed a relative risk of approximately 3 for Group 2 (HR = 3.033, 95% CI: 2.803–3.281, *p* < 0.001) and Group 3 (HR = 3.045, 95% CI: 2.874–3.226, *p* < 0.001), and around 5 for Group 4 (HR = 4.934, 95% CI: 4.403–5.529, *p* < 0.001) compared with Group 1. Interestingly, the interaction between AF and COPD on mortality risk was significantly negative (*p* for interaction < 0.001), meaning that the combined risk of both conditions was lower than the expected multiplication of their individual risks (see Supplemental Table S2).

### 3.5. Multivariable Analysis

The results of the multivariable model, which included the two investigated parameters (COPD and AF), besides the potential confounders, demonstrated an independent and statistically significant association of each with the outcome: adjHR = 1.672 (95% CI: 1.563–1.789, *p* < 0.001) and adjHR = 1.307 (95% CI: 1.236–1.381, *p* < 0.001) for COPD and AF, respectively (see Supplemental Table S3).

In the multivariate model (Table 2), which included the parameters of the study group and adjusted for potential confounders, the mortality risk of the patients from groups with either COPD, AF, or both was significantly higher compared with the reference group. However, in patients with both conditions (Group 4), the mortality risk was comparable to that of COPD alone, suggesting that AF did not further elevate the risk in the presence of COPD.

**Figure 2:**
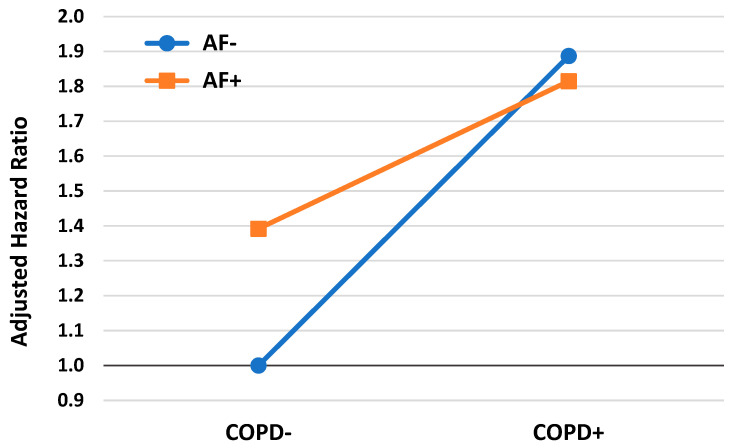
Adjusted relative risk for mortality by chronic obstructive pulmonary disease and atrial fibrillation—results of multivariable interactive model (see Supplemental Table S4). AF—Atrial fibrillation, COPD—Chronic obstructive pulmonary disease.

The results of the multivariable model with the parameter of interaction (COPD × AF) have shown that the negative interaction between AF and COPD in relation to the risk for long-term all-cause mortality was statistically significant (*p* for interaction < 0.001). The results of the interactive model are presented in Supplemental Table S4 and visually demonstrated in Figure 2.

### 3.6. Sub-Group Analysis

Among the patients with COPD, no significant association between AF and the outcome was found: AdjHR = 1.057 (95% CI: 0.919–1.215, *p* = 0.441). However, among the patients with no COPD, AF was significantly associated with the outcome: AdjHR = 1.371 (95% CI: 1.291–1.457, *p* < 0.001) (Supplemental Table S5). COPD was significantly associated with the outcome both for AF patients (AdjHR = 1.367, 95% CI: 1.206–1.549, *p* < 0.001) and for the patients with no AF (AdjHR = 1.876, 95% CI: 1.731–2.034, *p* < 0.001) (Supplemental Table S6). A subgroup analysis stratified by type of cardiovascular treatment showed that the associations between AF/COPD and long-term mortality remained similar and significant in both patients who underwent invasive treatment and those managed conservatively, with higher adjusted hazard ratios observed in the invasive treatment group (Supplemental Tables S7 and S8).

## 4. Discussion

This study leveraged a large AMI registry with long-term follow-up to examine the impact of COPD and AF on post-AMI outcomes. The key findings include the following: (1) a notable prevalence of COPD (8.9%) and AF (16.5%) among AMI survivors, with 2.4% having both conditions; (2) COPD and AF were linked to a higher burden of comorbidities, distinct clinical presentations, a more complicated in-hospital course, and a lower likelihood of undergoing revascularization; (3) univariate analysis revealed a threefold increase in mortality risk with either COPD or AF and a fivefold increase when both conditions coexisted; and (4) in the multivariate model, COPD (AdjHR = 1.9) and AF (AdjHR = 1.4) independently elevated mortality risk, but when both conditions were present, the mortality risk remained comparable to that of COPD alone (AdjHR = 1.8).

The significant prevalence of both AF and COPD in this study aligns with previous reports demonstrating an increased coexistence of these conditions. For instance, the prevalence of COPD among AF patients has varied across different registries, including 6.2% in the GLORIA-AF registry [9], 8.9% in the EORP-AF registry [23], and 13% in a large meta-analysis by Romiti et al. [8], which included 46 studies and over 4.2 million AF patients. Additionally, studies have reported a significantly higher prevalence of atrial arrhythmias, particularly AF, in patients with COPD, with rates reaching up to 29% [24,25,26]. Furthermore, in the Atherosclerosis Risk in Communities (ARIC) study, a decline in pulmonary function, as measured by forced expiratory volume in 1 s (FEV1), was associated with an increased incidence of AF [27]. Goedemans et al. [17] reported a higher prevalence of atrial arrhythmias in patients with COPD compared to those without COPD within one year following AMI. Additionally, the incidence of new-onset AF was twice as high in COPD patients (6%) as in non-COPD patients (3%). While rates of COPD and AF vary across contemporary acute coronary syndrome and AMI registries, they remain generally comparable to those found in the current study [28,29,30,31,32,33]. However, the strength of the current study lies in its routine assessment and dedicated focus on both comorbidities, their coexistence, and their interaction.

A more severe presentation, along with higher rates of in-hospital and short-term complications, has been reported in AMI patients with either COPD or AF [5,17,26,28,29,30,31,32,33,34,35]. Furthermore, we observed lower rates of revascularization following AMI in COPD and AF patients, consistent with previous reports. This has been speculated to be due to a higher burden of comorbidities, which increases procedural risks as well as diagnostic challenges in these patients [26,34,36]. However, most studies have assessed these conditions separately. Our study adds to the literature by evaluating presentation factors and complications based on the coexistence of both comorbidities. In addition, we demonstrated that the observed associations between AF/COPD and long-term mortality were present in both patients who underwent invasive treatment and those managed conservatively. Notably, several complications—such as cardiogenic shock, mechanical ventilation, blood transfusion, and pacing—were most prevalent in patients with both COPD and AF. This may be attributed to a higher burden of comorbidities in these patients, as well as an additive risk stemming directly from these conditions and potential interactions in their management.

One of the most significant findings of this study is the long-term outcomes of AMI survivors based on the presence of both AF and COPD. While both conditions have been individually linked to worse long-term outcomes, most prior analyses have either evaluated their coexistence outside of an AMI setting or assessed each condition separately in AMI survivors [5,17,26,28,29,30,31,32,33,36,37]. This study addresses that gap by specifically examining their combined impact post-AMI. In this context, we found that both COPD and AF were associated with significantly increased long-term mortality (HR = 3), which rose to HR = 5 when both conditions coexisted in the univariable analysis. However, after adjusting for potential confounders, while each condition independently elevated risk, the presence of both did not further increase mortality beyond that of COPD alone. This suggests that AF does not contribute to additional long-term risk when COPD is already present.

The heightened risk in AMI patients with COPD and AF is driven by complex, interrelated mechanisms. These likely include systemic processes common to both conditions, such as increased systemic inflammation, platelet hyperreactivity, coagulation factor dysregulation, elevated metalloproteinases (MMPs), hypoxia, oxidative stress and activation of the renin–angiotensin system, endothelial dysfunction, myocardial remodeling [13,26,38,39,40,41,42,43,44,45,46]. All these factors are particularly detrimental in this setting and may contribute to worse long-term outcomes [13,26,38,39,40,41].

The interaction between AF and COPD and their bidirectional detrimental effects, which further worsen post-AMI outcomes, can be attributed to several mechanisms. These include negative therapeutic interactions, particularly the underuse or misuse of anticoagulation and beta-blockers, as well as challenges with rhythm control therapies such as amiodarone and propafenone, which carry pulmonary side effects, or of other agents such as mineralocorticoid receptor antagonists (MRAs), which have been shown to reduce the risk of atrial fibrillation but may be selectively underused due to side effects, drug interactions, or other complexities [8,9,33,47,48,49,50,51,52,53,54,55]. Additionally, interventions like cardioversion, ablation, and revascularization are not only performed less frequently in these patients but also tend to have lower success rates. Furthermore, the overall increased clinical complexity, rate of recurrent exacerbations, and increased frailty of this population contribute to poorer outcomes [8,9,47,48,49,50,51,52,53,54,55].

A particularly interesting and unique finding of our study is that, following adjustment for confounders, while both COPD and AF were independently associated with increased long-term risk, the AF–COPD interaction on mortality was negative, indicating that the combined risk was lower than the expected product of their individual risks and that AF did not appear to further increase risk beyond that of COPD alone. Although no definitive explanation emerged, this could be due to specific characteristics within this group or analysis. Alternatively, COPD may serve as the dominant driver of long-term adverse outcomes in this setting, with much of the excess risk associated with AF being embedded within the overall risk conferred by COPD. AF may largely act as a marker of progressive COPD or underlying coronary artery disease, rather than adding significant independent risk. It is also possible that AF is at least partly triggered by high-dose beta agonists used for COPD treatment, rather than reflecting significant underlying substrate pathology or carrying substantial long-term prognostic implications [5]. Alternatively, it is possible that, following AMI, patients may have been more closely monitored and managed by cardiovascular-focused providers, thereby mitigating AF-associated risks more effectively than in patients with COPD.

This observation aligns with findings from a recent study by Warming et al. [10], which examined mortality risk based on the temporal sequence of COPD and AF diagnoses. Their study showed that when COPD was diagnosed before AF, it was associated with a higher mortality risk than when AF preceded COPD. Moreover, the earlier COPD was diagnosed relative to AF, the greater the excess mortality risk observed.

The key clinical implications of our findings highlight the importance of increased physician awareness in routinely assessing COPD and AF both immediately after an AMI admission and during long-term follow-up. Given the substantial short- and long-term risks associated with these comorbidities and their combination, proactive screening and management are essential. Additionally, a careful, individualized approach to treatment is crucial for secondary prevention of AMI in patients with either or both of these conditions. While potential treatment conflicts must be considered, it is equally important to avoid undertreatment. A multidisciplinary team of specialists, including those focused on cardiovascular disease and COPD, may be beneficial in developing a personalized, patient-centered management plan that optimally balances the risks and benefits of treatment strategies for both conditions. Importantly, the lack of interaction should not be interpreted as a reason to downplay AF in the setting of COPD, but rather as an indication that both conditions warrant thorough and serious management.

## 5. Limitations

This study is retrospective and observational, and as such, it shares the inherent limitations of this design. The relatively small sample size of patients with both AF and COPD may have constrained the identification of independent predictors in the multivariate analysis, potentially affecting the comprehensiveness of our findings. Additionally, we lacked data on AF subtypes (e.g., paroxysmal vs. chronic) and had no information on COPD validity and severity (e.g., Global Initiative for Chronic Obstructive Lung Disease [GOLD] stage, pulmonary function tests, and exacerbation history) or the temporal sequence of AF and COPD diagnoses, and whether this was preexisting or new-onset AF following AMI. The cohort includes patients from over two decades ago, during which the diagnosis and management of MI—including the availability and appropriateness of interventional treatments—have undergone substantial changes and therefore may not fully reflect contemporary patient populations. Furthermore, details regarding medical and interventional treatments for AF and COPD throughout the follow-up period were unavailable, potentially introducing bias and contributing to residual confounding.

## 6. Conclusions

This study provides insights into the long-term impact of COPD and AF following AMI. Both COPD and AF were associated with increased long-term mortality, with a three-fold higher risk for either condition alone or a fivefold higher risk when both coexisted in the univariable analysis. However, in multivariate analysis adjusted for confounders, AF did not further elevate risk beyond that of COPD alone, suggesting that COPD may be the dominant driver of adverse outcomes in this setting.

Patients with COPD and/or AF also experienced a more complex in-hospital course, were less likely to undergo revascularization, and had higher rates of short-term complications, particularly when both conditions coexisted. The findings highlight the importance of recognizing and managing these comorbidities as part of secondary prevention strategies for AMI. Given the potential for treatment conflicts and the risk of undertreatment, a multidisciplinary approach is crucial to optimizing patient outcomes.

Future research is warranted to further elucidate the mechanisms underlying the interaction between COPD and AF, as well as to explore targeted therapeutic strategies to mitigate their combined impact on post-AMI outcomes.

## Figures and Tables

**Table 1:** Baseline characteristics (at the index hospitalization) of the study population by study group.

Parameter	Value	Study Group				Total	*p*
		1 (COPD−, AF−)	2 (COPD+, AF−)	3 (COPD−, AF+)	4 (COPD+, AF+)		
*n*		11,892	1010	2171	376	15,449	
**Demographics**							
Age, years	Mean (SD)	63.38 (13.72) †	70.50 (11.11) #†	75.28 (11.38) #	75.42 (9.65) #	65.81 (13.94)	<0.001
	<65	6833 (57.5) †	312 (30.9) #†	402 (18.5) #	56 (14.9) #	7603 (49.2)	<0.001
	65–75	2478 (20.8) †	346 (34.3) #	544 (25.1) #†	123 (32.7) #	3491 (22.6)	
	≥75	2581 (21.7) †	352 (34.9) #†	1225 (56.4) #	197 (52.4) #	4355 (28.2)	
Sex	Male	8691 (73.1)	746 (73.9)	1188 (54.7) #†	266 (70.7)	10,891 (70.5)	<0.001
Ethnicity	Arab/other	2051 (17.2)	265 (26.2) #	215 (9.9) #†	75 (19.9)	2606 (16.9)	<0.001
**Cardiac diseases**							
Cardiomegaly		937 (7.9) †	163 (16.1) #†	368 (17.0) #†	100 (26.6) #	1568 (10.1)	<0.001
CHF		1816 (15.3) †	291 (28.8) #†	728 (33.5) #†	162 (43.1) #	2997 (19.4)	<0.001
Pulmonary heart disease		752 (6.3) †	184 (18.2) #†	460 (21.2) #†	137 (36.4) #	1533 (9.9)	<0.001
CIHD		10,023 (84.3)	836 (82.8)	1637 (75.4) #	305 (81.1)	12,801 (82.9)	<0.001
s/p MI		2118 (17.8) †	302 (29.9) #	542 (25.0) #†	131 (34.8) #	3093 (20.0)	<0.001
s/p PCI		2328 (19.6)	283 (28)	479 (22.1)	113 (30.1)	3203 (20.7)	<0.001
s/p CABG		997 (8.4) †	126 (12.5) #†	324 (14.9) #†	79 (21.0) #	1526 (9.9)	<0.001
AV block		410 (3.4) †	30 (3.0) †	96 (4.4)	27 (7.2) #	563 (3.6)	<0.001
**Cardiovascular risk factors**							
Chronic kidney disease		931 (7.8) †	126 (12.5) #†	355 (16.4) #	70 (18.6) #	1482 (9.6)	<0.001
Diabetes mellitus		4715 (39.6) †	517 (51.2) #	1004 (46.2) #	189 (50.3) #	6425 (41.6)	<0.001
Dyslipidemia		9924 (83.5) †	798 (79.0) #	1677 (77.2) #	292 (77.7) #	12,691 (82.1)	<0.001
Hypertension		6176 (51.9) †	564 (55.8)	1463 (67.4) #	239 (63.6) #	8442 (54.6)	<0.001
Obesity		2667 (22.4)	226 (22.4)	414 (19.1) #	83 (22.1)	3390 (21.9)	0.007
Smoking		5540 (46.6)	656 (65.0) #†	426 (19.6) #†	198 (52.7)	6820 (44.1)	<0.001
PVD		1195 (10.0) †	182 (18.0) #	356 (16.4) #	73 (19.4) #	1806 (11.7)	<0.001
Family history of IHD		1434 (12.1) †	51 (5.0) #	74 (3.4) #	7 (1.9) #	1566 (10.1)	<0.001
**Other disorders**							
Neurological disorders		1748 (14.7) †	183 (18.1) #	565 (26.0) #	84 (22.3) #	2580 (16.7)	<0.001
Malignancy		447 (3.8)	50 (5.0)	117 (5.4) #	24 (6.4)	638 (4.1)	<0.001
Anemia		4846 (40.8) †	534 (52.9) #†	1229 (56.6) #	236 (62.8) #	6845 (44.3)	<0.001
GI bleeding		206 (1.7) †	24 (2.4) †	83 (3.8) #	21 (5.6) #	334 (2.2)	<0.001
Schizophrenia/Psychosis		171 (1.4)	24 (2.4)	46 (2.1)	6 (1.6)	247 (1.6)	0.023
Alcohol/drug addiction		245 (2.1)	44 (4.4) #	22 (1.0) #†	10 (2.7)	321 (2.1)	<0.001
History of malignancy		575 (4.8) †	75 (7.4) #	180 (8.3) #	37 (9.8) #	867 (5.6)	<0.001
**Characteristics of AMI**							
Type of AMI	STEMI	5679 (47.8) †	311 (30.8) #	593 (27.3) #	90 (23.9) #	6673 (43.2)	<0.001
Admitted/transposed to ICCU		8238 (69.3) †	527 (52.2) #†	984 (45.3) #	150 (39.9) #	9899 (64.1)	<0.001
Length of hospital stay, days	Mean (SD)	9.30 (8.81) †	10.88 (9.81) #†	11.73 (11.18) #†	13.53 (15.82) #	9.85 (9.53)	<0.001
	≥7	5057 (42.5) †	519 (51.4) #	1187 (54.7) #	211 (56.1) #	6974 (45.1)	<0.001
**Type of treatment**	Noninvasive	2798 (23.5) †	405 (40.1) #†	1043 (48.0) #†	220 (58.5) #	4466 (28.9)	<0.001
	PCI	7378 (62.0) †	502 (49.7) #†	913 (42.1) #	134 (35.6) #	8927 (57.8)	
	CABG	1716 (14.4) †	103 (10.2) #	215 (9.9) #	22 (5.9) #	2056 (13.3)	
**Acute in-hospital events**							
Cardiac arrest		36 (0.3)	4 (0.4)	12 (0.6)	2 (0.5)	54 (0.3)	0.291
Cardiogenic shock		180 (1.5)	23 (2.3)	70 (3.2) #	12 (3.2)	285 (1.8)	<0.001
Intra-aortic balloon pulsation		257 (2.2)	13 (1.3)	61 (2.8)	6 (1.6)	337 (2.2)	0.038
Any form of pacing		201 (1.7) †	16 (1.6) †	74 (3.4) #	19 (5.1) #	310 (2.0)	<0.001
Mechanical ventilation		368 (3.1) †	69 (6.8) #	122 (5.6) #†	39 (10.4) #	598 (3.9)	<0.001
Blood transfusion		1413 (11.9) †	136 (13.5)	353 (16.3) #	71 (18.9) #	1973 (12.8)	<0.001
Sepsis		92 (0.8)	15 (1.5)	37 (1.7) #	6 (1.6)	150 (1)	<0.001
**Results of echocardiography**							
Echocardiography performance	*n*	8780	617	1305	206	10,908	
Severe LV dysfunction		908 (10.3) †	105 (17.0) #	224 (17.2) #	43 (20.9) #	1280 (11.7)	<0.001
LV hypertrophy		398 (4.5) †	40 (6.5)	112 (8.6) #	21 (10.2) #	571 (5.2)	<0.001
Mitral regurgitation		339 (3.9) †	41 (6.6) #†	159 (12.2) #	28 (13.6) #	567 (5.2)	<0.001
Tricuspid regurgitation		177 (2.0) †	26 (4.2) #†	150 (11.5) #	27 (13.1) #	380 (3.5)	<0.001
Pulmonary hypertension		420 (4.8) †	80 (13.0) #†	247 (18.9) #†	56 (27.2) #	803 (7.4)	<0.001
**Results of angiography**							
Angiography performance	*n*	8074	538	993	148	9753	
Measure of CAD	No/non-significant	354 (4.4)	40 (7.4) #	105 (10.6) #	10 (6.8)	509 (5.2)	<0.001
	One vessel	2358 (29.2)	122 (22.7) #	196 (19.7) #	33 (22.3)	2709 (27.8)	<0.001
	Two vessels	2304 (28.5)	150 (27.9)	229 (23.1) #	44 (29.7)	2727 (28.0)	<0.001
	Three vessels/LM	3058 (37.9)	226 (42.0)	463 (46.6) #	61 (41.2)	3808 (39.0)	<0.001

Data are presented as numbers (percentage), unless specified otherwise. AF—Atrial fibrillation, AMI—Acute myocardial infarction, AV—Atrioventricular, CABG—Coronary artery bypass grafting, CAD—Coronary artery disease, CHF—Congestive heart failure, CIHD—Chronic ischemic heart disease, COPD—Chronic obstructive pulmonary disease, GI—Gastro-intestinal, ICCU—Intensive Cardiac Care Unit, IHD—Ischemic heart disease, LM—Left main artery, LV—Left ventricular, MI—Myocardial infarction, PCI—Percutaneous coronary intervention, PVD—Peripheral vascular disease, s/p—Status post, STEMI—ST-elevation myocardial infarction. #—Statistical significance (*p* < 0.05) compared with Group 1 (COPD−, AF−). †—Statistical significance (*p* < 0.05) compared with Group 4 (COPD+, AF+).

**Table 2:** Relative risk for mortality—multivariable analysis.

Parameter	Values	B (SE)	AdjHR	(95% CI)	*p*
Study group	1 (COPD−, AF−)		1 (ref.)		
	2 (COPD+, AF−)	0.635 (0.041)	1.887	(1.742; 2.045)	<0.001
	3 (COPD−, AF+)	0.331 (0.031)	1.392	(1.311; 1.479)	<0.001
	4 (COPD+, AF+)	0.596 (0.060)	1.815	(1.614; 2.040)	<0.001
Age, years	<65		1 (ref.)		
	65–75	0.814 (0.037)	2.258	(2.100; 2.428)	<0.001
	≥75	1.303 (0.037)	3.681	(3.425; 3.956)	<0.001
Sex	Male vs. Female	−0.121 (0.027)	0.886	(0.841; 0.934)	<0.001
CHF		0.325 (0.028)	1.385	(1.310; 1.463)	<0.001
s/p MI		0.160 (0.028)	1.173	(1.110; 1.240)	<0.001
Chronic kidney disease		0.453 (0.033)	1.573	(1.474; 1.680)	<0.001
Diabetes mellitus		0.276 (0.025)	1.318	(1.254; 1.386)	<0.001
Dyslipidemia		−0.181 (0.030)	0.834	(0.787; 0.884)	<0.001
Obesity		−0.135 (0.033)	0.874	(0.819; 0.932)	<0.001
PVD		0.338 (0.032)	1.403	(1.317; 1.494)	<0.001
Neurological disorders		0.421 (0.028)	1.524	(1.443; 1.609)	<0.001
Malignancy		0.573 (0.047)	1.774	(1.617; 1.946)	<0.001
Anemia		0.327 (0.027)	1.387	(1.315; 1.463)	<0.001
Alcohol/drug addiction		0.755 (0.082)	2.128	(1.811; 2.500)	<0.001
Type of AMI	NSTEMI vs. STEMI	0.174 (0.028)	1.191	(1.126; 1.258)	<0.001
Type of treatment	Noninvasive		1 (ref.)		
	PCI	−0.682 (0.052)	0.506	(0.456; 0.560)	<0.001
	CABG	−1.110 (0.061)	0.329	(0.292; 0.371)	<0.001
Mechanical ventilation		0.109 (0.053)	1.115	(1.005; 1.238)	0.041
Blood transfusion		0.140 (0.036)	1.150	(1.071; 1.235)	<0.001
Severe LV dysfunction		0.386 (0.042)	1.471	(1.357; 1.596)	<0.001
LV hypertrophy		0.246 (0.059)	1.279	(1.140; 1.436)	<0.001
Pulmonary hypertension		0.275 (0.045)	1.317	(1.206; 1.439)	<0.001
Measure of CAD	No/non-significant/One vessel		1 (ref.)		
	Two vessels	0.118 (0.052)	1.125	(1.016; 1.246)	0.024
	Three vessels/LM	0.322 (0.047)	1.380	(1.259; 1.512)	<0.001

AdjHR—Adjusted hazard ratio, AF—Atrial fibrillation, AMI—Acute myocardial infarction, B—regression coefficient, CABG—Coronary artery bypass grafting, CAD—Coronary artery disease, CHF—Congestive heart failure, CI—Confidence interval, COPD—Chronic obstructive pulmonary disease, LM—Left main artery, LV—Left ventricular, MI—Myocardial infarction, NSTEMI—non-ST-elevation myocardial infarction, PCI—Percutaneous coronary intervention, PVD—Peripheral vascular disease, ref.—Reference group, SE—Standard error, s/p—Status post, STEMI—ST-elevation myocardial infarction.

## Data Availability

The data underlying this article may be shared upon reasonable request to the corresponding author.

## Supplementary Materials

The following supporting information can be downloaded at https://www.mdpi.com/article/10.3390/jcm14165907/s1: Figure S1: Study flowchart., Table S1: Baseline characteristics of the study population, Figure S2: Cumulative survival functions for post-acute myocardial infarction all-cause mortality through-out the follow-up period up to 10 years, Table S2: Relationships between chronic obstructive pulmonary disease, atrial fibrillation, and between the risk for post-acute myocardial infarction all-cause mortality through-out the follow-up period up to 10 years—interaction analysis., Table S3: Relationships between chronic obstructive pulmonary disease, atrial fibrillation, and between the risk for post-acute myocardial infarction all-cause mortality through-out the follow-up period up to 10 years—multivariable analysis., Table S4: Relationships between chronic obstructive pulmonary disease, atrial fibrillation, and between the risk for post-acute myocardial infarction all-cause mortality through-out the follow-up period up to 10 years—multivariable interaction analysis., Table S5: Relationships between atrial fibrillation and the risk for post-acute myocardial infarction all-cause mortality through-out the follow-up period up to 10 years—multivariable analysis, Table S6: Relationships between chronic obstructive pulmonary disease and the risk for post-acute myocardial infarction all-cause mortality through-out the follow-up period up to 10 years—multivariable analysis, Table S7: All-cause mortality following AMI stratified by chronic obstructive pulmonary disease/atrial fibrillation status and type of cardiovascular intervention—multivariable analysis, Table S8: All-cause mortality following AMI stratified by chronic obstructive pulmonary disease/atrial fibrillation status and type of cardiovascular intervention—multivariable interaction analysis.

## Abbreviations

AF	Atrial fibrillation
AMI	Acute myocardial infarction
CABG	Coronary artery bypass grafting
CAD	Coronary artery disease
COPD	Chronic obstructive pulmonary disease
ECG	Electrocardiogram
FEV1	Forced expiratory volume in 1 s
HR	Hazard ratio
ICD-9-CM	International Classification of Diseases, Ninth Revision, Clinical Modification
IQR	Interquartile range
LV	Left ventricular
MMPs	Metalloproteinases
NSTEMI	Non-ST-elevation myocardial infarction
PCI	Percutaneous coronary intervention
STEMI	ST-elevation myocardial infarction
