# Supplementary material for: Long-Term Impact of Chronic Obstructive Pulmonary Disease and Atrial Fibrillation on Post-Acute Myocardial Infarction Long-Term All-Cause Mortality: Insights from the SAMI III Project
Source: J Clin Med. 2025 Aug 21;14(16):5907. doi: 10.3390/jcm14165907 (PMC12387317; doi:10.3390/jcm14165907)
Supplement: Supplementary file 1 [file jcm-14-05907-s001.zip › jcm-3802201-supplementary.pdf]

Supplemental materials

Supplemental Figure S1. Study flowchart.

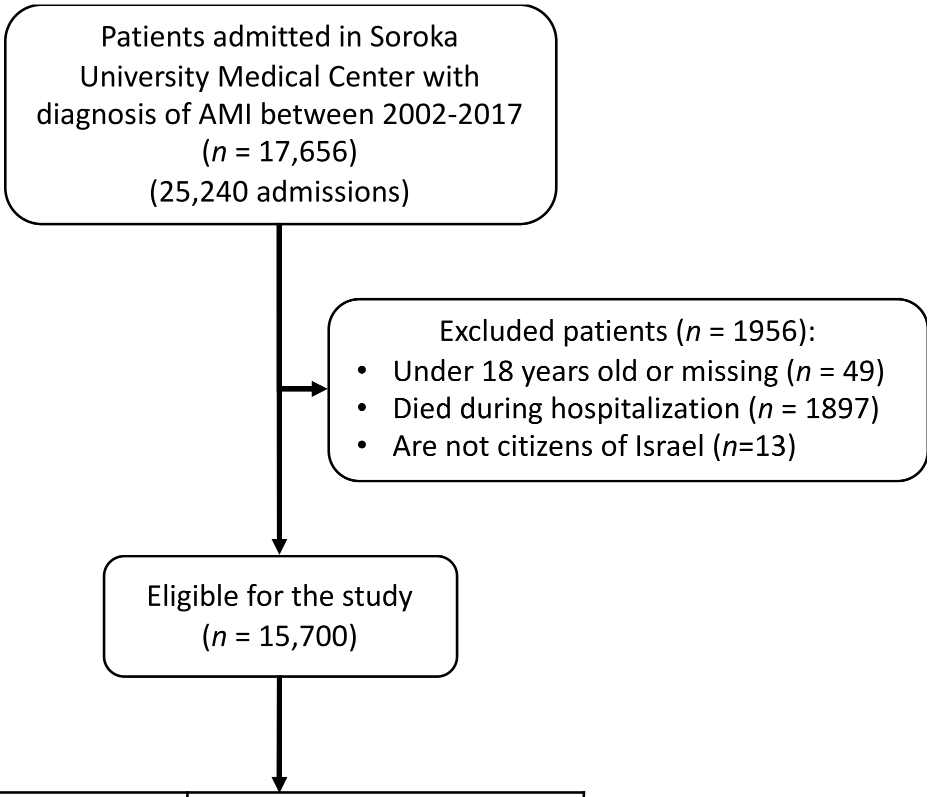

		AF		
		No	Yes	Total
COPD	No	11,870	2404	14,274
	Yes	1003	423	1426
	Total	12,873	2827	15,700

AMI—Acute myocardial infarction, COPD—Chronic obstructive pulmonary disease, AF—Atrial fibrillation.

Supplemental Table S1. Baseline characteristics of the study population:

A) By chronic obstructive pulmonary disease status.

Parameter	Value	No COPD	COPD	Total	*p*
n		14,063	1386	15,449	
**Demographics**					
Age, years	Mean (SD)	65.22 (14.06)	71.83 (10.95)	65.81 (9.85)	<0.001
	<65	7235 (51.4)	368 (26.6)	7603 (49.2)	<0.001
	65-75	3022 (21.5)	469 (33.8)	3491 (22.6)	
	≥75	3806 (27.1)	549 (39.6)	4355 (28.2)	
Sex	Male	9879 (70.2)	1012 (73.0)	10,891 (70.5)	0.031
Ethnicity	Arab/other	2266 (16.1)	340 (24.5)	2606 (16.9)	<0.001
**Cardiac diseases**					
Cardiomegaly		1305 (9.3)	263 (19.0)	1568 (10.1)	<0.001
CHF		2544 (18.1)	453 (32.7)	2997 (19.4)	<0.001
Pulmonary heart disease		1212 (8.6)	321 (23.2)	1533 (9.9)	<0.001
CIHD		11,660 (82.9)	1141 (82.3)	12,801 (82.9)	0.579
s/p MI		2660 (18.9)	433 (31.2)	3093 (20)	<0.001
s/p PCI		2807 (20.0)	396 (28.6)	3203 (20.7)	<0.001
s/p CABG		1321 (9.4)	205 (14.8)	1526 (9.9)	<0.001
AV block		506 (3.6)	57 (4.1)	563 (3.6)	0.329
**Cardiovascular risk factors**					
Chronic kidney disease		1286 (9.1)	196 (14.1)	1482 (9.6)	<0.001
Diabetes mellitus		5719 (40.7)	706 (50.9)	6425 (41.6)	<0.001
Dyslipidemia		11,601 (82.5)	1090 (78.6)	12,691 (82.1)	<0.001
Hypertension		7639 (54.3)	803 (57.9)	8442 (54.6)	0.010
Obesity		3081 (21.9)	309 (22.3)	3390 (21.9)	0.741
Smoking		5966 (42.4)	854 (61.6)	6820 (44.1)	<0.001
PVD		1551 (11.0)	255 (18.4)	1806 (11.7)	<0.001
Family history of IHD		1508 (10.7)	58 (4.2)	1566 (10.1)	<0.001
**Other disorders**					
Neurological disorders		2313 (16.4)	267 (19.3)	2580 (16.7)	0.007
Malignancy		564 (4.0)	74 (5.3)	638 (4.1)	0.018
Anemia		6075 (43.2)	770 (55.6)	6845 (44.3)	<0.001
GI bleeding		289 (2.1)	45 (3.2)	334 (2.2)	0.004
Schizophrenia/Psychosis		217 (1.5)	30 (2.2)	247 (1.6)	0.078
Alcohol/drug addiction		267 (1.9)	54 (3.9)	321 (2.1)	<0.001
History of malignancy		755 (5.4)	112 (8.1)	867 (5.6)	<0.001
**Characteristics of AMI**					
Type of AMI	STEMI	6272 (44.6)	401 (28.9)	6673 (43.2)	<0.001
Admitted/transposed to ICCU		9222 (65.6)	677 (48.8)	9899 (64.1)	<0.001
Length of hospital stay, days	Mean (SD)	9.67 (9.26)	11.60 (11.80)	9.85 (9.53)	<0.001

	≥7	6244 (44.4)	730 (52.7)	6974 (45.1)	<0.001
**Type of treatment**	Noninvasive	3841 (27.3)	625 (45.1)	4466 (28.9)	<0.001
	PCI	8291 (59.0)	636 (45.9)	8927 (57.8)	
	CABG	1931 (13.7)	125 (9.0)	2056 (13.3)	
**Acute in-hospital events**					
Cardiac arrest		48 (0.3)	6 (0.4)	54 (0.3)	0.582
Cardiogenic shock		250 (1.8)	35 (2.5)	285 (1.8)	0.048
Intra-aortic balloon pulsation		318 (2.3)	19 (1.4)	337 (2.2)	0.030
Any form of pacing		275 (2.0)	35 (2.5)	310 (2.0)	0.149
Mechanical ventilation		490 (3.5)	108 (7.8)	598 (3.9)	<0.001
Blood transfusion		1766 (12.6)	207 (14.9)	1973 (12.8)	0.011
Sepsis		129 (0.9)	21 (1.5)	150 (1.0)	0.030
**Results of echocardiography**					
Echocardiography performance	*n*	10,085	823	10,908	
Severe LV dysfunction		1132 (11.2)	148 (18.0)	1280 (11.7)	<0.001
LV hypertrophy		510 (5.1)	61 (7.4)	571 (5.2)	0.004
Mitral regurgitation		498 (4.9)	69 (8.4)	567 (5.2)	<0.001
Tricuspid regurgitation		327 (3.2)	53 (6.4)	380 (3.5)	<0.001
Pulmonary hypertension		667 (6.6)	136 (16.5)	803 (7.4)	<0.001
**Results of angiography**					
Angiography performance	*n*	9067	686	9753	
Measure of CAD	No/non-significant	459 (5.1)	50 (7.3)	509 (5.2)	0.002
	One vessel	2554 (28.2)	155 (22.6)	2709 (27.8)	
	Two vessels	2533 (27.9)	194 (28.3)	2727 (28.0)	
	Three vessels/LM	3521 (38.8)	287 (41.8)	3808 (39.0)	

B) By atrial fibrillation status.

**Parameter**	**Value**	**No AF**	**AF**	**Total**	***p***
n		**12,902**	**2547**	**15,449**	
**Demographics**					
Age, years	Mean (SD)	63.94 (13.67)	75.30 (11.14)	65.81 (9.85)	<0.001
	<65	7145 (55.4)	458 (18.0)	7603 (49.2)	<0.001
	65-75	2824 (21.9)	667 (26.2)	3491 (22.6)	
	≥75	2933 (22.7)	1422 (55.8)	4355 (28.2)	
Sex	Male	9437 (73.1)	1454 (57.1)	10,891 (70.5)	<0.001
Ethnicity	Arab/other	2316 (18.0)	290 (11.4)	2606 (16.9)	<0.001
**Cardiac diseases**					
Cardiomegaly		1100 (8.5)	468 (18.4)	1568 (10.1)	<0.001
CHF		2107 (16.3)	890 (34.9)	2997 (19.4)	<0.001
Pulmonary heart disease		936 (7.3)	597 (23.4)	1533 (9.9)	<0.001
CIHD		10,859 (84.2)	1942 (76.2)	12,801 (82.9)	<0.001
s/p MI		2420 (18.8)	673 (26.4)	3093 (20)	<0.001

s/p PCI		2611 (20.2)	592 (23.2)	3203 (20.7)	<0.001
s/p CABG		1123 (8.7)	403 (15.8)	1526 (9.9)	<0.001
AV block		440 (3.4)	123 (4.8)	563 (3.6)	<0.001
**Cardiovascular risk factors**					
Chronic kidney disease		1057 (8.2)	425 (16.7)	1482 (9.6)	<0.001
Diabetes mellitus		5232 (40.6)	1193 (46.8)	6425 (41.6)	<0.001
Dyslipidemia		10,722 (83.1)	1969 (77.3)	12,691 (82.1)	<0.001
Hypertension		6740 (52.2)	1702 (66.8)	8442 (54.6)	<0.001
Obesity		2893 (22.4)	497 (19.5)	3390 (21.9)	0.001
Smoking		6196 (48)	624 (24.5)	6820 (44.1)	<0.001
PVD		1377 (10.7)	429 (16.8)	1806 (11.7)	<0.001
Family history of IHD		1485 (11.5)	81 (3.2)	1566 (10.1)	<0.001
**Other disorders**					
Neurological disorders		1931 (15)	649 (25.5)	2580 (16.7)	<0.001
Malignancy		497 (3.9)	141 (5.5)	638 (4.1)	<0.001
Anemia		5380 (41.7)	1465 (57.5)	6845 (44.3)	<0.001
GI bleeding		230 (1.8)	104 (4.1)	334 (2.2)	<0.001
Schizophrenia/Psychosis		195 (1.5)	52 (2.0)	247 (1.6)	0.051
Alcohol/drug addiction		289 (2.2)	32 (1.3)	321 (2.1)	0.001
History of malignancy		650 (5.0)	217 (8.5)	867 (5.6)	<0.001
**Characteristics of AMI**					
Type of AMI	STEMI	5990 (46.4)	683 (26.8)	6673 (43.2)	<0.001
Admitted/transposed to ICCU		8765 (67.9)	1134 (44.5)	9899 (64.1)	<0.001
Length of hospital stay, days	Mean (SD)	9.42 (8.91)	12.00 (11.99)	9.85 (9.53)	<0.001
	≥7	5576 (43.2)	1398 (54.9)	6974 (45.1)	<0.001
**Type of treatment**	Noninvasive	3203 (24.8)	1263 (49.6)	4466 (28.9)	<0.001
	PCI	7880 (61.1)	1047 (41.1)	8927 (57.8)	
	CABG	1819 (14.1)	237 (9.3)	2056 (13.3)	
**Acute in-hospital events**					
Cardiac arrest		40 (0.3)	14 (0.5)	54 (0.3)	0.061
Cardiogenic shock		203 (1.6)	82 (3.2)	285 (1.8)	<0.001
Intra-aortic balloon pulsation		270 (2.1)	67 (2.6)	337 (2.2)	0.089
Any form of pacing		217 (1.7)	93 (3.7)	310 (2.0)	<0.001
Mechanical ventilation		437 (3.4)	161 (6.3)	598 (3.9)	<0.001
Blood transfusion		1549 (12.0)	424 (16.6)	1973 (12.8)	<0.001
Sepsis		107 (0.8)	43 (1.7)	150 (1.0)	<0.001
**Results of echocardiography**					
Echocardiography performance	*n*	9397	1511	10,908	
Severe LV dysfunction		1013 (10.8)	267 (17.7)	1280 (11.7)	<0.001
LV hypertrophy		438 (4.7)	133 (8.8)	571 (5.2)	<0.001
Mitral regurgitation		380 (4.0)	187 (12.4)	567 (5.2)	<0.001
Tricuspid regurgitation		203 (2.2)	177 (11.7)	380 (3.5)	<0.001
Pulmonary hypertension		500 (5.3)	303 (20.1)	803 (7.4)	<0.001

**Results of angiography**					
Angiography performance	*n*	8612	1141	9753	
Measure of CAD	No/non-significant	394 (4.6)	115 (10.1)	509 (5.2)	<0.001
	One vessel	2480 (28.8)	229 (20.1)	2709 (27.8)	
	Two vessels	2454 (28.5)	273 (23.9)	2727 (28.0)	
	Three vessels/LM	3284 (38.1)	524 (45.9)	3808 (39.0)	

Data are presented as numbers (percentage), unless specified otherwise. AF—Atrial fibrillation, AMI—Acute myocardial infarction, AV—Atrioventricular, CABG—Coronary artery bypass grafting, CAD—Coronary artery disease, CHF—Congestive heart failure, CIHD—Chronic ischemic heart disease, COPD—Chronic obstructive pulmonary disease, GI—Gastro-intestinal, ICCU—Intensive Cardiac Care Unit, IHD—Ischemic heart disease, LM—Left main artery, LV—Left ventricular, MI—Myocardial infarction, PCI—Percutaneous coronary intervention, PVD—Peripheral vascular disease, s/p—Status post, STEMI—ST-elevation myocardial infarction.

Supplemental Figure S2. Cumulative survival functions for post-acute myocardial infarction all-cause mortality through-out the follow-up period up to 10 years:

A) By chronic obstructive pulmonary disease status.

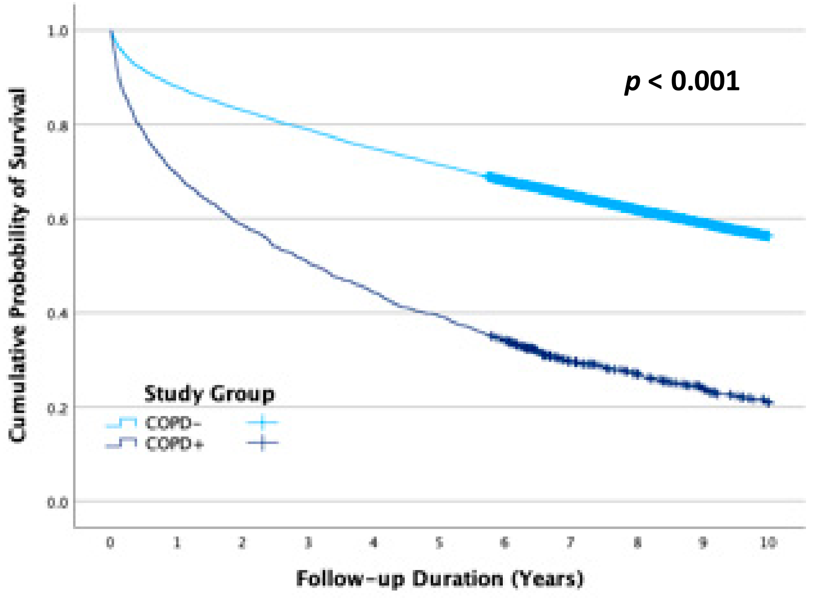

B) By atrial fibrillation status.

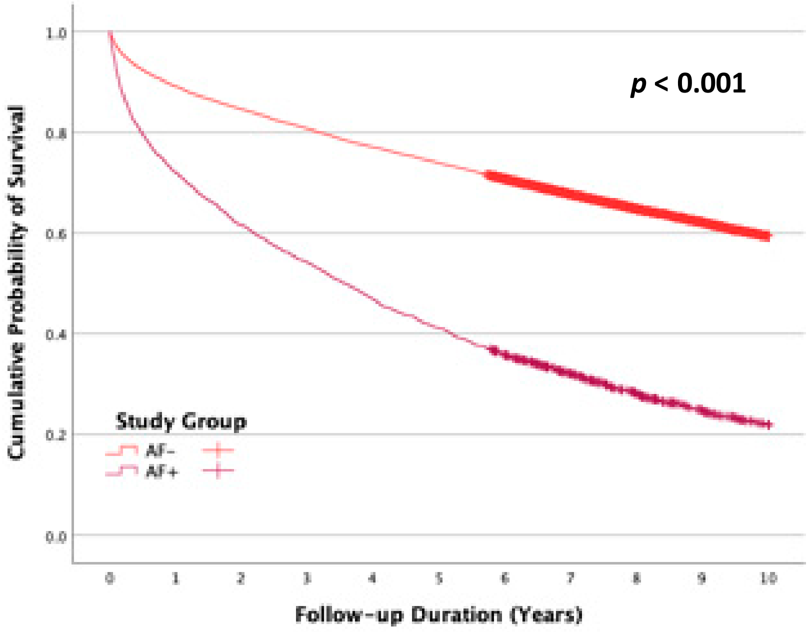

AF—Atrial fibrillation, COPD—Chronic obstructive pulmonary disease.

Supplemental Table S2. Relationships between chronic obstructive pulmonary disease, atrial fibrillation, and between the risk for post-acute myocardial infarction all-cause mortality through-out the follow-up period up to 10 years – interaction analysis.

**Values**	**B (SE)**	**AdjHR**	**(95% CI)**	***p***
COPD	1.109 (0.040)	3.033	(2.803; 3.2815)	<0.001
AF	1.113 (0.029)	3.045	(2.874; 3.226)	<0.001
COPD × AF[#]	-0.627 (0.073)	0.534	(0.463; 0.617)	<0.001

AdjHR—Adjusted hazard ratio, AF—Atrial fibrillation, B—regression coefficient, CI—Confidence interval, COPD—Chronic obstructive pulmonary disease, SE—Standard error. #—Interaction parameter.

Supplemental Table S3. Relationships between chronic obstructive pulmonary disease, atrial fibrillation, and between the risk for post-acute myocardial infarction all-cause mortality through-out the follow-up period up to 10 years – multivariable analysis.

Parameter	Values	B (SE)	AdjHR	(95% CI)	*p*
COPD		0.514 (0.035)	1.672	(1.563; 1.789)	<0.001
AF		0.267 (0.028)	1.307	(1.236; 1.381)	<0.001
Age, years	<65		1 (ref.)		
	65-75	0.824 (0.037)	2.279	(2.119; 2.45)	<0.001
	≥75	1.313 (0.037)	3.717	(3.459; 3.995)	<0.001
Sex	Male vs Female	-0.126 (0.027)	0.882	(0.837; 0.929)	<0.001
CHF		0.322 (0.028)	1.380	(1.306; 1.459)	<0.001
s/p MI		0.155 (0.028)	1.167	(1.105; 1.234)	<0.001
Chronic kidney disease		0.451 (0.033)	1.569	(1.470; 1.675)	<0.001
Diabetes mellitus		0.278 (0.025)	1.321	(1.257; 1.388)	<0.001
Dyslipidemia		-0.183 (0.030)	0.833	(0.786; 0.883)	<0.001
Obesity		-0.133 (0.033)	0.875	(0.821; 0.933)	<0.001
PVD		0.341 (0.032)	1.406	(1.320; 1.497)	<0.001
Neurological disorders		0.417 (0.028)	1.517	(1.437; 1.602)	<0.001
Malignancy		0.576 (0.047)	1.779	(1.622; 1.952)	<0.001
Anemia		0.326 (0.027)	1.386	(1.314; 1.461)	<0.001
Alcohol/drug addiction		0.768 (0.082)	2.155	(1.834; 2.532)	<0.001
Type of AMI	NSTEMI vs STEMI	0.176 (0.028)	1.192	(1.127; 1.261)	<0.001
Type of treatment	Noninvasive		1 (ref.)		
	PCI	-0.682 (0.052)	0.505	(0.456; 0.56)	<0.001
	CABG	-1.111 (0.061)	0.329	(0.292; 0.371)	<0.001
Mechanical Ventilation		0.099 (0.053)	1.104	(0.994; 1.225)	0.064
Blood Transfusion		0.140 (0.036)	1.150	(1.071; 1.235)	<0.001
Severe LV dysfunction		0.388 (0.042)	1.473	(1.358; 1.598)	<0.001
LV hypertrophy		0.241 (0.059)	1.273	(1.134; 1.429)	<0.001
Pulmonary hypertension		0.272 (0.045)	1.312	(1.201; 1.434)	<0.001
Measure of CAD	No/non-significant/One vessel		1 (ref.)		
	Two vessels	0.117 (0.052)	1.124	(1.015; 1.245)	0.025
	Three vessels/LM	0.323 (0.047)	1.381	(1.260; 1.513)	<0.001

AdjHR—Adjusted hazard ratio, AF—Atrial fibrillation, AMI—Acute myocardial infarction, B—regression coefficient, CABG—Coronary artery bypass grafting, CAD—Coronary artery disease, CHF—Congestive heart failure, CI—Confidence interval, COPD—Chronic obstructive pulmonary disease, LM—Left main artery, LV—Left ventricular, MI—Myocardial infarction, NSTEMI—non-ST-elevation myocardial infarction, PCI—Percutaneous coronary intervention, PVD—Peripheral vascular disease, ref.—Reference group, SE—Standard error, s/p—Status post, STEMI—ST-elevation myocardial infarction.

Supplemental Table S4. Relationships between chronic obstructive pulmonary disease, atrial fibrillation, and between the risk for post-acute myocardial infarction all-cause mortality through-out the follow-up period up to 10 years – multivariable interaction analysis.

Parameter	Values	B (SE)	AdjHR	(95% CI)	*p*
COPD		0.635 (0.041)	1.887	(1.742; 2.045)	<0.001
AF		0.331 (0.031)	1.392	(1.311; 1.479)	<0.001
COPD × AF#		-0.370 (0.073)	0.691	(0.598; 0.797)	<0.001
Age, years	<65		1 (ref.)		
	65-75	0.814 (0.037)	2.258	(2.100; 2.428)	<0.001
	≥75	1.303 (0.037)	3.681	(3.425; 3.956)	<0.001
Sex	Male vs Female	-0.121 (0.027)	0.886	(0.841; 0.934)	<0.001
CHF		0.325 (0.028)	1.385	(1.310; 1.463)	<0.001
s/p MI		0.160 (0.028)	1.173	(1.110; 1.240)	<0.001
Chronic kidney disease		0.453 (0.033)	1.573	(1.474; 1.680)	<0.001
Diabetes mellitus		0.276 (0.025)	1.318	(1.254; 1.386)	<0.001
Dyslipidemia		-0.181 (0.030)	0.834	(0.787; 0.884)	<0.001
Obesity		-0.135 (0.033)	0.874	(0.819; 0.932)	<0.001
PVD		0.338 (0.032)	1.403	(1.317; 1.494)	<0.001
Neurological disorders		0.421 (0.028)	1.524	(1.443; 1.609)	<0.001
Malignancy		0.573 (0.047)	1.774	(1.617; 1.946)	<0.001
Anemia		0.327 (0.027)	1.387	(1.315; 1.463)	<0.001
Alcohol/drug addiction		0.755 (0.082)	2.128	(1.811; 2.500)	<0.001
Type of AMI	NSTEMI vs STEMI	0.174 (0.028)	1.191	(1.126; 1.258)	<0.001
Type of treatment	Noninvasive		1 (ref.)		
	PCI	-0.682 (0.052)	0.506	(0.456; 0.560)	<0.001
	CABG	-1.110 (0.061)	0.329	(0.292; 0.371)	<0.001
Mechanical Ventilation		0.109 (0.053)	1.115	(1.005; 1.238)	0.041
Blood Transfusion		0.140 (0.036)	1.150	(1.071; 1.235)	<0.001
Severe LV dysfunction		0.386 (0.042)	1.471	(1.357; 1.596)	<0.001
LV hypertrophy		0.246 (0.059)	1.279	(1.140; 1.436)	<0.001
Pulmonary hypertension		0.275 (0.045)	1.317	(1.206; 1.439)	<0.001
Measure of CAD	No/non-significant/One vessel		1 (ref.)		
	Two vessels	0.118 (0.052)	1.125	(1.016; 1.246)	0.024
	Three vessels/LM	0.322 (0.047)	1.380	(1.259; 1.512)	<0.001

AdjHR—Adjusted hazard ratio, AF—Atrial fibrillation, AMI—Acute myocardial infarction, B—regression coefficient, CABG—Coronary artery bypass grafting, CAD—Coronary artery disease, CHF—Congestive heart failure, CI—Confidence interval, COPD—Chronic obstructive pulmonary disease, LM—Left main artery, LV—Left ventricular, MI—Myocardial infarction, NSTEMI—non-ST-elevation myocardial infarction, PCI—Percutaneous coronary intervention, PVD—Peripheral vascular disease, ref.—Reference group, SE—Standard error, s/p—Status post, STEMI—ST-elevation myocardial infarction. #—Interaction parameter

Supplemental Table S5. Relationships between atrial fibrillation and the risk for post-acute myocardial infarction all-cause mortality through-out the follow-up period up to 10 years – multivariable analysis:

A) Among the patients with chronic obstructive pulmonary disease.

Parameter	Values	B (SE)	AdjHR	(95% CI)	*p*
AF		0.055 (0.071)	1.057	(0.919; 1.215)	0.441
Age, years	<65		1 (ref.)		
	65-75	0.525 (0.094)	1.690	(1.405; 2.034)	<0.001
	≥75	0.818 (0.097)	2.266	(1.874; 2.741)	<0.001
Sex	Male vs Female	-0.038 (0.074)	0.963	(0.833; 1.113)	0.608
CHF		0.277 (0.070)	1.320	(1.150; 1.514)	<0.001
s/p MI		0.109 (0.069)	1.116	(0.974; 1.277)	0.113
Chronic kidney disease		0.305 (0.088)	1.356	(1.141; 1.612)	<0.001
Diabetes mellitus		0.205 (0.066)	1.227	(1.078; 1.397)	0.002
Dyslipidemia		-0.070 (0.077)	0.932	(0.802; 1.085)	0.364
Obesity		-0.168 (0.084)	0.845	(0.718; 0.996)	0.045
PVD		0.264 (0.080)	1.303	(1.114; 1.523)	<0.001
Neurological disorders		0.232 (0.077)	1.262	(1.085; 1.467)	0.003
Malignancy		0.134 (0.131)	1.143	(0.884; 1.478)	0.306
Anemia		0.127 (0.072)	1.135	(0.986; 1.307)	0.077
Alcohol/drug addiction		0.472 (0.167)	1.604	(1.156; 2.225)	0.005
Type of AMI	NSTEMI vs STEMI	0.116 (0.076)	1.124	(0.967; 1.306)	0.128
Type of treatment	Noninvasive		1 (ref.)		
	PCI	-0.478 (0.137)	0.620	(0.474; 0.810)	<0.001
	CABG	-1.145 (0.175)	0.318	(0.226; 0.449)	<0.001
Mechanical Ventilation		0.133 (0.112)	1.143	(0.918; 1.423)	0.234
Blood Transfusion		0.316 (0.095)	1.372	(1.139; 1.652)	<0.001
Severe LV dysfunction		0.360 (0.106)	1.433	(1.164; 1.763)	<0.001
LV hypertrophy		0.014 (0.159)	1.014	(0.742; 1.385)	0.931
Pulmonary hypertension		0.263 (0.108)	1.301	(1.052; 1.609)	0.015
Measure of CAD	No/non-significant/One vessel		1 (ref.)		
	Two vessels	-0.095 (0.131)	0.909	(0.703; 1.176)	0.467
	Three vessels/LM	0.025 (0.120)	1.025	(0.811; 1.297)	0.835

B) Among the patients with no chronic obstructive pulmonary disease.

Parameter	Values	B (SE)	AdjHR	(95% CI)	*p*
AF		0.316 (0.031)	1.371	(1.291; 1.457)	<0.001
Age, years	<65		1 (ref.)		
	65-75	0.834 (0.040)	2.302	(2.128; 2.491)	<0.001
	≥75	1.365 (0.040)	3.914	(3.622; 4.230)	<0.001
Sex	Male vs Female	-0.123 (0.029)	0.885	(0.836; 0.935)	<0.001
CHF		0.332 (0.031)	1.393	(1.312; 1.480)	<0.001
s/p MI		0.169 (0.031)	1.184	(1.115; 1.258)	<0.001
Chronic kidney disease		0.475 (0.036)	1.609	(1.499; 1.727)	<0.001
Diabetes mellitus		0.276 (0.028)	1.318	(1.249; 1.392)	<0.001
Dyslipidemia		-0.196 (0.032)	0.822	(0.772; 0.876)	<0.001
Obesity		-0.130 (0.036)	0.878	(0.819; 0.942)	<0.001
PVD		0.351 (0.035)	1.420	(1.325; 1.522)	<0.001
Neurological disorders		0.444 (0.030)	1.558	(1.469; 1.653)	<0.001
Malignancy		0.685 (0.051)	1.984	(1.796; 2.192)	<0.001
Anemia		0.367 (0.029)	1.443	(1.362; 1.529)	<0.001
Alcohol/drug addiction		0.784 (0.095)	2.19	(1.818; 2.638)	<0.001
Type of AMI	NSTEMI vs STEMI	0.185 (0.031)	1.203	(1.133; 1.277)	<0.001
Type of treatment	Noninvasive		1 (ref.)		
	PCI	-0.706 (0.057)	0.494	(0.442; 0.552)	<0.001
	CABG	-1.107 (0.066)	0.330	(0.290; 0.376)	<0.001
Mechanical Ventilation		0.112 (0.061)	1.119	(0.993; 1.261)	0.066
Blood Transfusion		0.110 (0.040)	1.116	(1.033; 1.206)	0.005
Severe LV dysfunction		0.388 (0.045)	1.474	(1.349; 1.611)	<0.001
LV hypertrophy		0.282 (0.064)	1.326	(1.171; 1.502)	<0.001
Pulmonary hypertension		0.273 (0.050)	1.314	(1.192; 1.449)	<0.001
Measure of CAD	No/non-significant/One vessel		1 (ref.)		
	Two vessels	0.148 (0.057)	1.159	(1.037; 1.296)	0.009
	Three vessels/LM	0.377 (0.051)	1.458	(1.320; 1.611)	<0.001

AdjHR—Adjusted hazard ratio, AF—Atrial fibrillation, AMI—Acute myocardial infarction, B—regression coefficient, CABG—Coronary artery bypass grafting, CAD—Coronary artery disease, CHF—Congestive heart failure, CI—Confidence interval, LM—Left main artery, LV—Left ventricular, MI—Myocardial infarction, NSTEMI—non-ST-elevation myocardial infarction, PCI—Percutaneous coronary intervention, PVD—Peripheral vascular disease, ref.—Reference group, SE—Standard error, s/p—Status post, STEMI—ST-elevation myocardial infarction.

Supplemental Table S6. Relationships between chronic obstructive pulmonary disease and the risk for post-acute myocardial infarction all-cause mortality throughout the follow-up period up to 10 years – multivariable analysis:

A) Among the patients with atrial fibrillation.

Parameter	Values	B (SE)	AdjHR	(95% CI)	*p*
COPD		0.313 (0.064)	1.367	(1.206; 1.549)	<0.001
Age, years	<65		1 (ref.)		
	65-75	0.644 (0.089)	1.905	(1.601; 2.267)	<0.001
	≥75	1.032 (0.085)	2.808	(2.378; 3.316)	<0.001
Sex	Male vs Female	-0.155 (0.050)	0.856	(0.776; 0.945)	0.002
CHF		0.236 (0.049)	1.266	(1.149; 1.395)	<0.001
s/p MI		0.130 (0.053)	1.139	(1.027; 1.262)	0.013
Chronic kidney disease		0.345 (0.061)	1.412	(1.253; 1.590)	<0.001
Diabetes mellitus		0.143 (0.048)	1.154	(1.051; 1.268)	0.003
Dyslipidemia		-0.088 (0.055)	0.916	(0.822; 1.021)	0.114
Obesity		-0.063 (0.062)	0.939	(0.832; 1.060)	0.312
PVD		0.279 (0.060)	1.321	(1.175; 1.486)	<0.001
Neurological disorders		0.312 (0.051)	1.367	(1.235; 1.512)	<0.001
Malignancy		0.654 (0.095)	1.924	(1.597; 2.317)	<0.001
Anemia		0.263 (0.051)	1.301	(1.178; 1.438)	<0.001
Alcohol/drug addiction		0.934 (0.204)	2.544	(1.706; 3.795)	<0.001
Type of AMI	NSTEMI vs STEMI	0.170 (0.058)	1.185	(1.058; 1.328)	0.003
Type of treatment	Noninvasive		1 (ref.)		
	PCI	-0.567 (0.102)	0.567	(0.464; 0.692)	<0.001
	CABG	-0.894 (0.123)	0.409	(0.321; 0.520)	<0.001
Mechanical Ventilation		0.081 (0.092)	1.085	(0.905; 1.300)	0.379
Blood Transfusion		0.059 (0.067)	1.060	(0.929; 1.210)	0.384
Severe LV dysfunction		0.207 (0.081)	1.230	(1.050; 1.442)	0.010
LV hypertrophy		0.273 (0.105)	1.314	(1.070; 1.613)	0.009
Pulmonary hypertension		0.272 (0.073)	1.313	(1.137; 1.516)	<0.001
Measure of CAD	No/non-significant/One vessel		1 (ref.)		
	Two vessels	0.109 (0.110)	1.115	(0.898; 1.384)	0.324
	Three vessels/LM	0.345 (0.094)	1.412	(1.175; 1.697)	<0.001

B) Among the patients with no atrial fibrillation.

Parameter	Values	B (SE)	AdjHR	(95% CI)	*p*
COPD		0.629 (0.041)	1.876	(1.731; 2.034)	<0.001
Age, years	<65		1 (ref.)		
	65-75	0.814 (0.041)	2.257	(2.082; 2.446)	<0.001
	≥75	1.346 (0.041)	3.840	(3.544; 4.161)	<0.001
Sex	Male vs Female	-0.106 (0.031)	0.900	(0.846; 0.957)	<0.001
CHF		0.364 (0.034)	1.439	(1.346; 1.539)	<0.001
s/p MI		0.176 (0.034)	1.193	(1.117; 1.274)	<0.001
Chronic kidney disease		0.509 (0.040)	1.663	(1.537; 1.799)	<0.001
Diabetes mellitus		0.323 (0.030)	1.381	(1.301; 1.465)	<0.001
Dyslipidemia		-0.219 (0.035)	0.804	(0.750; 0.861)	<0.001
Obesity		-0.167 (0.039)	0.846	(0.784; 0.913)	<0.001
PVD		0.360 (0.038)	1.433	(1.329; 1.545)	<0.001
Neurological disorders		0.457 (0.033)	1.580	(1.480; 1.686)	<0.001
Malignancy		0.521 (0.055)	1.683	(1.511; 1.874)	<0.001
Anemia		0.339 (0.032)	1.403	(1.317; 1.495)	<0.001
Alcohol/drug addiction		0.716 (0.090)	2.045	(1.713; 2.442)	<0.001
Type of AMI	NSTEMI vs STEMI	0.162 (0.033)	1.177	(1.104; 1.255)	<0.001
Type of treatment	Noninvasive		1 (ref.)		
	PCI	-0.715 (0.061)	0.489	(0.434; 0.552)	<0.001
	CABG	-1.175 (0.071)	0.309	(0.268; 0.355)	<0.001
Mechanical Ventilation		0.111 (0.066)	1.117	(0.982; 1.270)	0.093
Blood Transfusion		0.184 (0.044)	1.202	(1.104; 1.310)	<0.001
Severe LV dysfunction		0.437 (0.049)	1.548	(1.407; 1.703)	<0.001
LV hypertrophy		0.217 (0.072)	1.243	(1.080; 1.430)	0.002
Pulmonary hypertension		0.253 (0.058)	1.287	(1.150; 1.442)	<0.001
Measure of CAD	No/non-significant/One vessel		1 (ref.)		
	Two vessels	0.117 (0.059)	1.125	(1.001; 1.264)	0.048
	Three vessels/LM	0.297 (0.054)	1.346	(1.211; 1.496)	<0.001

AdjHR—Adjusted hazard ratio, AMI—Acute myocardial infarction, B—regression coefficient, CABG—Coronary artery bypass grafting, CAD—Coronary artery disease, CHF—Congestive heart failure, CI—Confidence interval, COPD—Chronic obstructive pulmonary disease, LM—Left main artery, LV—Left ventricular, MI—Myocardial infarction, NSTEMI—non-ST-elevation myocardial infarction, PCI—Percutaneous coronary intervention, PVD—Peripheral vascular disease, ref.—Reference group, SE—Standard error, s/p—Status post, STEMI—ST-elevation myocardial infarction.

Supplemental Table S7. All-cause mortality following AMI stratified by chronic obstructive pulmonary disease/atrial fibrillation status and invasive cardiovascular intervention – multivariable analysis:

A) Among the patients that underwent invasive cardiovascular intervention (percutaneous coronary intervention/coronary artery bypass grafting).

Parameter	Values	B (SE)	AdjHR	(95% CI)	*p*
Study group	1 (COPD−, AF−)		1 (ref.)		
	2 (COPD+, AF−)	0.799 (0.058)	2.224	(1.985; 2.492)	<0.001
	3 (COPD−, AF+)	0.445 (0.046)	1.560	(1.425; 1.708)	<0.001
	4 (COPD+, AF+)	0.703 (0.099)	2.019	(1.663; 2.452)	<0.001
Age, years	<65		1 (ref.)		
	65-75	0.837 (0.046)	2.308	(2.108; 2.528)	<0.001
	≥75	1.363 (0.048)	3.908	(3.556; 4.295)	<0.001
Sex	Male vs Female	-0.148 (0.040)	0.863	(0.798; 0.932)	<0.001
CHF		0.382 (0.043)	1.465	(1.347; 1.593)	<0.001
s/p MI		0.224 (0.040)	1.251	(1.156; 1.354)	<0.001
Chronic kidney disease		0.702 (0.053)	2.018	(1.819; 2.239)	<0.001
Diabetes mellitus		0.383 (0.037)	1.467	(1.365; 1.576)	<0.001
Dyslipidemia		-0.179 (0.048)	0.836	(0.761; 0.918)	<0.001
Obesity		-0.110 (0.042)	0.896	(0.826; 0.972)	0.008
PVD		0.366 (0.046)	1.442	(1.317; 1.579)	<0.001
Neurological disorders		0.408 (0.043)	1.504	(1.382; 1.637)	<0.001
Malignancy		0.705 (0.075)	2.024	(1.748; 2.342)	<0.001
Anemia		0.316 (0.038)	1.372	(1.273; 1.478)	<0.001
Alcohol/drug addiction		0.864 (0.106)	2.373	(1.928; 2.919)	<0.001
Type of AMI	NSTEMI vs STEMI	0.209 (0.037)	1.233	(1.147; 1.326)	<0.001
Mechanical Ventilation		0.227 (0.075)	1.255	(1.084; 1.454)	0.002
Blood Transfusion		-0.104 (0.049)	0.901	(0.819; 0.991)	0.031
Severe LV dysfunction		0.408 (0.049)	1.503	(1.364; 1.656)	<0.001
LV hypertrophy		0.318 (0.073)	1.374	(1.190; 1.586)	<0.001
Pulmonary hypertension		0.247 (0.059)	1.281	(1.141; 1.438)	<0.001
Measure of CAD	No/non-significant/One vessel		1 (ref.)		
	Two vessels	0.068 (0.053)	1.070	(0.965; 1.188)	0.200
	Three vessels/LM	0.189 (0.048)	1.208	(1.100; 1.326)	<0.001

B) Among the patients that did not undergo invasive treatment.

Parameter	Values	B (SE)	AdjHR	(95% CI)	*p*
Study group	1 (COPD−, AF−)		1 (ref.)		
	2 (COPD+, AF−)	0.486 (0.058)	1.626	(1.451; 1.822)	<0.001
	3 (COPD−, AF+)	0.226 (0.041)	1.254	(1.157; 1.358)	<0.001
	4 (COPD+, AF+)	0.489 (0.075)	1.630	(1.407; 1.889)	<0.001
Age, years	<65		1 (ref.)		
	65-75	0.689 (0.062)	1.992	(1.763; 2.251)	<0.001
	≥75	1.142 (0.058)	3.134	(2.798; 3.509)	<0.001
Sex	Male vs Female	-0.088 (0.036)	0.916	(0.854; 0.983)	0.014
CHF		0.284 (0.038)	1.329	(1.234; 1.431)	<0.001
s/p MI		0.119 (0.039)	1.127	(1.043; 1.217)	0.003
Chronic kidney disease		0.318 (0.043)	1.374	(1.264; 1.494)	<0.001
Diabetes mellitus		0.165 (0.036)	1.179	(1.100; 1.264)	<0.001
Dyslipidemia		-0.159 (0.038)	0.853	(0.792; 0.919)	<0.001
Obesity		-0.204 (0.054)	0.816	(0.734; 0.907)	<0.001
PVD		0.318 (0.045)	1.374	(1.258; 1.501)	<0.001
Neurological disorders		0.386 (0.037)	1.470	(1.369; 1.580)	<0.001
Malignancy		0.469 (0.061)	1.598	(1.417; 1.802)	<0.001
Anemia		0.293 (0.038)	1.340	(1.243; 1.445)	<0.001
Alcohol/drug addiction		0.599 (0.132)	1.821	(1.407; 2.356)	<0.001
Type of AMI	NSTEMI vs STEMI	0.094 (0.043)	1.099	(1.010; 1.196)	0.029
Mechanical Ventilation		0.020 (0.077)	1.020	(0.877; 1.187)	0.798
Blood Transfusion		0.170 (0.049)	1.185	(1.075; 1.305)	<0.001
Severe LV dysfunction		0.254 (0.079)	1.289	(1.105; 1.505)	0.001
LV hypertrophy		0.150 (0.100)	1.162	(0.955; 1.413)	0.134
Pulmonary hypertension		0.283 (0.071)	1.327	(1.155; 1.525)	<0.001
Measure of CAD	No/non-significant/One vessel		1 (ref.)		
	Two vessels	0.748 (0.414)	2.112	(0.939; 4.752)	0.071
	Three vessels/LM	0.762 (0.370)	2.143	(1.038; 4.423)	0.039

AdjHR—Adjusted hazard ratio, AF—Atrial fibrillation, AMI—Acute myocardial infarction, B—regression coefficient, CAD—Coronary artery disease, CHF—Congestive heart failure, CI—Confidence interval, COPD—Chronic obstructive pulmonary disease, LM—Left main artery, LV—Left ventricular, MI—Myocardial infarction, NSTEMI—non-ST-elevation myocardial infarction, PVD—Peripheral vascular disease, ref.—Reference group, SE—Standard error, s/p—Status post, STEMI—ST-elevation myocardial infarction.

Supplemental Table 8. All-cause mortality following AMI stratified by chronic obstructive pulmonary disease/atrial fibrillation status and type of cardiovascular intervention – multivariable interaction analysis:

A) Among the patients that underwent invasive cardiovascular intervention (percutaneous coronary intervention/coronary artery bypass grafting).

Parameter	Values	B (SE)	AdjHR	(95% CI)	*p*
COPD		0.799 (0.058)	2.224	(1.985; 2.492)	<0.001
AF		0.445 (0.046)	1.560	(1.425; 1.708)	<0.001
COPD × AF[#]		-0.541 (0.117)	0.582	(0.462; 0.732)	<0.001
Age, years	<65		1 (ref.)		
	65-75	0.837 (0.046)	2.308	(2.108; 2.528)	<0.001
	≥75	1.363 (0.048)	3.908	(3.556; 4.295)	<0.001
Sex	Male vs Female	-0.148 (0.040)	0.863	(0.798; 0.932)	<0.001
CHF		0.382 (0.043)	1.465	(1.347; 1.593)	<0.001
s/p MI		0.224 (0.040)	1.251	(1.156; 1.354)	<0.001
Chronic kidney disease		0.702 (0.053)	2.018	(1.819; 2.239)	<0.001
Diabetes mellitus		0.383 (0.037)	1.467	(1.365; 1.576)	<0.001
Dyslipidemia		-0.179 (0.048)	0.836	(0.761; 0.918)	<0.001
Obesity		-0.110 (0.042)	0.896	(0.826; 0.972)	0.008
PVD		0.366 (0.046)	1.442	(1.317; 1.579)	<0.001
Neurological disorders		0.408 (0.043)	1.504	(1.382; 1.637)	<0.001
Malignancy		0.705 (0.075)	2.024	(1.748; 2.342)	<0.001
Anemia		0.316 (0.038)	1.372	(1.273; 1.478)	<0.001
Alcohol/drug addiction		0.864 (0.106)	2.373	(1.928; 2.919)	<0.001
Type of AMI	NSTEMI vs STEMI	0.209 (0.037)	1.233	(1.147 ;1.326)	<0.001
Mechanical Ventilation		0.227 (0.075)	1.255	(1.084; 1.454)	0.002
Blood Transfusion		-0.104 (0.049)	0.901	(0.819; 0.991)	0.031
Severe LV dysfunction		0.408 (0.049)	1.503	(1.364; 1.656)	<0.001
LV hypertrophy		0.318 (0.073)	1.374	(1.190; 1.586)	<0.001
Pulmonary hypertension		0.247 (0.059)	1.281	(1.141; 1.438)	<0.001
Measure of CAD	No/non-significant/One vessel		1 (ref.)		
	Two vessels	0.068 (0.053)	1.070	(0.965; 1.188)	0.200
	Three vessels/LM	0.189 (0.048)	1.208	(1.100; 1.326)	<0.001

B) Among the patients that did not undergo invasive cardiovascular treatment.

Parameter	Values	B (SE)	AdjHR	(95% CI)	*p*
COPD		0.486 (0.058)	1.626	(1.451; 1.822)	<0.001
AF		0.226 (0.041)	1.254	(1.157; 1.358)	<0.001
COPD × AF[#]		-0.223 (0.096)	0.800	(0.662; 0.966)	0.021
Age, years	<65		1 (ref.)		
	65-75	0.689 (0.062)	1.992	(1.763; 2.251)	<0.001
	≥75	1.1420 (0.058)	3.134	(2.798; 3.509)	<0.001
Sex	Male vs Female	-0.088 (0.036)	0.916	(0.854; 0.983)	0.014
CHF		0.284 (0.038)	1.329	(1.234; 1.431)	<0.001
s/p MI		0.119 (0.039)	1.127	(1.043; 1.217)	0.003
Chronic kidney disease		0.318 (0.043)	1.374	(1.264; 1.494)	<0.001
Diabetes mellitus		0.165 (0.036)	1.179	(1.100; 1.264)	<0.001
Dyslipidemia		-0.159 (0.038)	0.853	(0.792; 0.919)	<0.001
Obesity		-0.204 (0.054)	0.816	(0.734; 0.907)	<0.001
PVD		0.318 (0.045)	1.374	(1.258; 1.501)	<0.001
Neurological disorders		0.386 (0.037)	1.470	(1.369; 1.580)	<0.001
Malignancy		0.469 (0.061)	1.598	(1.417; 1.802)	<0.001
Anemia		0.293 (0.038)	1.340	(1.243; 1.445)	<0.001
Alcohol/drug addiction		0.599 (0.132)	1.821	(1.407; 2.356)	<0.001
Type of AMI	NSTEMI vs STEMI	0.094 (0.043)	1.099	(1.010 ;1.196)	0.029
Mechanical Ventilation		0.020 (0.077)	1.020	(0.877; 1.187)	0.798
Blood Transfusion		0.170 (0.049)	1.185	(1.075; 1.305)	<0.001
Severe LV dysfunction		0.254 (0.079)	1.289	(1.105; 1.505)	0.001
LV hypertrophy		0.150 (0.100)	1.162	(0.955; 1.413)	0.134
Pulmonary hypertension		0.283 (0.071)	1.327	(1.155; 1.525)	<0.001
Measure of CAD	No/non-significant/One vessel		1 (ref.)		
	Two vessels	0.748 (0.414)	2.112	(0.939; 4.752)	0.071
	Three vessels/LM	0.762 (0.370)	2.143	(1.038; 4.423)	0.039

AdjHR—Adjusted hazard ratio, AF—Atrial fibrillation, AMI—Acute myocardial infarction, B—regression coefficient, CAD—Coronary artery disease, CHF—Congestive heart failure, CI—Confidence interval, COPD—Chronic obstructive pulmonary disease, LM—Left main artery, LV—Left ventricular, MI—Myocardial infarction, NSTEMI—non-ST-elevation myocardial infarction, PVD—Peripheral vascular disease, ref.—Reference group, SE—Standard error, s/p—Status post, STEMI—ST-elevation myocardial infarction. #—Interaction parameter.
